# Molecular detection of *Staphylococcus aureus* in urine in patients with *S. aureus* bacteremia: an exploratory study

**DOI:** 10.1007/s10096-024-04969-7

**Published:** 2024-10-31

**Authors:** Franziska Schuler, Achim J. Kaasch, Frieder Schaumburg

**Affiliations:** 1 Institute of Medical Microbiology, University Hospital Münster, Münster, Germany; 2 Institute of Medical Microbiology and Hospital Hygiene, Otto von Guericke University Magdeburg, Magdeburg, Germany

**Keywords:** *Staphylococcus aureus*, Bacteremia, Bacteriuria, Nucleic acid amplification test, Rapid assay, Urine

## Abstract

**Purpose:**

*Staphylococcus aureus* bacteremia (SAB) is associated with a 90-day mortality of 28–34%. Many SAB-patients (7.8–39%) have a secondary *S. aureus* bacteriuria (SABU) mainly without symptoms of a urinary tract infection. Due to high morbidity and mortality, there is an interest in rapid detection of *S. aureus* bacteremia. Here, we compared a rapid nucleic acid amplification test (NAAT) with conventional culture to detect S. *aureus* in urine and to identify cases with increased risk for SAB.

**Methods:**

In a cross-sectional study, we assessed urine samples (mid-stream, clean catch and catheter urine) of patients with SAB and bacteremia other than SAB (non-SAB). Urine samples were collected ± 3 days to the collection of the positive blood culture and were cultured on a set of selective and non-selective agar plates. NAAT was performed using a commercial test (Xpert^®^ SA Nasal Complete G3, Cepheid) from a sterile swab soaked in urine.

**Results:**

We included samples from 100 patients (68% male, median age: 67.4 years) with SAB and 20 patients (75% male, median age: 65.84 years) with non-SAB. The sensitivity of detecting SAB from urine samples was 47% (specificity: 90%) for NAAT, when applying a Ct-value of ≤ 37.4 for positive results. Urine culture had a sensitivity of 25% and a specificity of 95%. Molecular and culture methods showed a moderate agreement (80%, Cohens kappa: 0.55).

**Conclusion:**

NAAT from urine has a higher sensitivity than culture in patients with SAB and could potentially identify cases with increased risk for SAB. Future studies should investigate whether this characteristic could translate into a clinical benefit through rapid detection of SAB.

**Supplementary Information:**

The online version contains supplementary material available at 10.1007/s10096-024-04969-7.

## Introduction

*Staphylococcus aureus* bacteremia (SAB) is common with an annual incidence of 17–38/100,000 residents [1–4]. Despite antibiotic therapy, SAB has a high 90-day mortality (28–34%) [5–7].

For the empirical therapy of an infection with unknown focus and/or without pathogen identification, cephalosporins with or without vancomycin are frequently chosen. Compared to the staphylococcal-specific beta-lactams (e.g. flucloxacillin, cefazolin), the empirical therapy with cephalosporins (e.g. cefuroxime, ceftriaxone/cefotaxime) or beta-lactam-beta-lactamase combinations is associated with a higher 30-day mortality of SAB in a retrospective study [8]. Several studies have described lower survival rates of SAB-patients with a methicillin-susceptible isolate under vancomycin therapy [9–11]. In addition, secondary foci may occur in 16–34% of patients as a complication of SAB [7, 12]. Treatment delay was strongly associated with the presence of metastatic foci, but not associated with mortality [13]. Since early, specific therapy is important for a favourable outcome of SAB from an antibiotic stewardship and antimicrobial resistance (AMR) view, there is a clinical need for a rapid diagnosis of SAB.

True *S. aureus* urinary tract infections in our setting are presumably uncommon as we detect *S. aureus* in urine in only 1.4–1.9% of all positive cultures from clinical samples [14]. Worldwide, many SAB patients (7.8–39%) have concomitant *S. aureus* bacteriuria (SABU) [15–25]. The urinary tract as the primary source of SAB is infrequent (3.2%, *n* = 132/4181 SAB patients) [26] and is seen almost exclusively in the context of inserted foreign bodies (e.g. indwelling catheters) or urologic interventions [15, 26]. SABU secondary to SAB is usually asymptomatic (e.g. absence of dysuria, flank or suprapubic pain, gross haematuria). The pathomechanisms of secondary SABU in patients with SAB are not fully understood and might include tissue destruction, micro-abscesses, transcytosis, paracytosis, or intracellular translocation in leukocytes and macrophages [25].

Currently, the state-of-the-art in microbiological diagnostics of bloodstream infections is species identification with matrix-assisted laser desorption/ionization time-of-flight mass spectrometry (MALDI-TOF MS) performed directly from positive blood cultures or from short sub-cultures on solid medium. Additionally, multiplex-polymerase chain reaction (PCR) from blood targeting the most common species can achieve a shorter turn-around time (e.g. 3.5–6 h) [27]. The species identification from blood cultures is usually available on the same day the blood culture becomes positive. The median time of blood cultures to become positive is roughly 21 h after collection of the sample including slow growing pathogens [28].

Urine is readily available, and we hypothesize that detecting secondary SABU with a nucleic acid amplification test (NAAT) may enable a more rapid detection (within one hour) than urine culture. However, the diagnostic accuracy of such an approach to identify SAB from urine has not been assessed yet. Therefore, the objective of this study was to compare the test performance of a rapid NAAT with conventional urine culture to detect *S. aureus* in urine and evaluate the clinical benefit through rapid detection of SAB.

## Methods

### Ethical consideration

Ethical approval was obtained from the institutional review board (IRB) of the University of Münster (Ethik-Kommission Westfalen-Lippe, 2020-615-f-S). The IRB granted a waiver to obtain a signed written informed consent from patients.

### Patients

This cross-sectional study was carried out at a tertiary care hospital in Germany (~ 1,300 beds) between February 2020 and May 2023. Specimen of the patients were sent for clinical routine. Patients were included consecutively if they had a culture-confirmed bacteremia and a urine culture ± 3 days to the collection of the positive blood culture. Patients with a likely contamination of the blood culture (e.g. coagulase negative staphylococci in one out of four blood culture bottles without signs of infection) were excluded. For each patient, age, sex and primary focus of infection were recorded.

### Microbiological culture

Mid-stream, clean catch or catheter urine of patients with bacteremia was collected in a sterile tube (Urin-Monovette^®^, Sarstedt, Nümbrecht, Germany). Urine samples (10 µl per plate) were cultured on Columbia Blood agar with 5% Sheep Blood (BD, Heidelberg, Germany; ambient air), MacConkey agar (BD, ambient air) and a selective agar for Gram-positive bacteria (Columbia CNA Agar with 5% Sheep Blood, BD, 5% CO_2_) for a maximum of 48 h at 35 ± 2 °C. We defined SABU as detection of *S. aureus* in urine independent of the concentration (in colony forming units (CFU)/ml) [29].

Pairs of aerobic and anaerobic blood culture bottles (BD) were incubated in a Bactec^®^ FX device (BD) for five days (14 days in case of suspected endocarditis) and were subcultured on Columbia Blood agar (BD) and/or chocolate agar (BD, 5% CO_2_) when the blood culture bottles were flagged positive [30]. Species identification was done with MALDI-TOF MS (Biotyper^®^ Sirius one, Bruker, Bremen, Germany) applying the Biotyper software IVD/Compass (version 4.2).

A screening test was used to detect substances that could inhibit the growth of bacteria in urine (e.g. antibiotics, cytostatic drugs). For that purpose, a blank cellulose disc (ThermoFisher scientific, Wesel, Germany) soaked with 10 µl urine was placed on a Mueller-Hinton II agar (BD, 35 ± 2 °C, ambient air) inoculated with a highly susceptible bacterium (ATCC 6031 *Bacillus subtilis*). If an inhibition zone around the disc was seen after 24 h (ambient air, 35 ± 2 °C), the test was interpreted as positive. This screening was recommended in German guidelines until 2020 to aid the interpretation of negative urine cultures in patients with a high suspicion of urinary tract infection.

### Nucleic acid amplification test for *S. aureus*

After urine culture had been carried out urine samples were stored at -20 °C until NAAT was done to guarantee equal bacterial cell counts for culture and NAAT. For NAAT, we used the Xpert^®^ SA Nasal Complete G3 test cartridge (Cepheid, Krefeld, Germany) which was developed and validated for rapid (within one hour) and simultaneous detection of *S. aureus* and methicillin-resistant *S. aureus* (MRSA) in nasal swab samples. The Xpert^®^ SA Nasal Complete Assay is a qualitative test for in-vitro diagnostics. The primers and probes detect simultaneously proprietary sequences of the staphylococcal protein A (*spa*), *mecA* and SCC*mec* inserted into the *S. aureus* chromosome at the *attB* site.

For the current study, we soaked a sterile swab (eSwab, Copan, Brescia, Italy) with urine, washed it in the buffer solution of the test cartridge and removed it afterwards. Then, we performed the test as recommended for nasal swabs. This procedure was chosen to use the test as recommended by the manufacturer with the only variation that urine instead of nasal swabs was sampled.

### Analytical sensitivity

The limit of detection (LoD) is defined as the lowest number of CFU per sample that can be distinguished from negative samples. We selected urine with negative inhibitory test to test the LoD. We calculated the mean volume of urine that can be squeezed out of the swabs into the elution buffer of the cartridge test (= sample per test). Starting from a stock concentration of 2.45 × 10^8^/ml of ATCC 29,213 *S. aureus*, we prepared a dilution series in urine for NAAT. Since the calculated CFU/sample of a dilution series only gives an estimate of the real CFU/sample, we cultured the bacterial suspension of the dilution series on Columbia Blood agar (24 h, 35 ± 2 °C, ambient air) and used the cultured bacterial counts to calculate the LoD.

### Statistical analysis

A specific sample size calculation was not done in the absence of any data on the performance of the NAAT in urine samples. We considered a samples size of 100 SAB and 20 non-SAB cases as appropriate for our objective applying convenience sampling. Differences between groups in categorical variables were compared using Fisher´s exact test or Chi-squared test (RStudio version 4.3.1; [31]) with a significance level of < 0.05.

To define the optimal Ct-threshold for the prediction of SAB, we performed a receiver operating characteristic (ROC) analysis using the Ct value as the predictor and SAB as the outcome. For samples with undetermined Ct values (i.e. no detection after 40 cycles), we imputed a Ct value and set a maximum value of 40. The ROC curves and optimal cut-off values (Youden-Index) were computed with the R package “cutpointr” [32].

The test performance of NAAT and culture detection to predict SAB was calculated (sensitivity, specificity) using the optimal cut-off Ct value as determined by the ROC analysis. The concordance of molecular and culture test was calculated using Cohen’s kappa coefficient.

## Results

We included 100 patients (68% male, median age: 67.4 years [range 0.6–93.2]) with SAB and 20 patients (75% male, median age: 65.8 years [range 30.3–83.3]) with positive blood cultures other than *S. aureus* (non-SAB). The most common infective focus in patients with SAB was the central venous catheter (20%, 20/100), skin and soft tissue infections (16%, 16/100), pneumonia (10%, 10/100), endocarditis (6%, 6/100) or remained unknown (24%, 24/100).

**Fig. 1:**
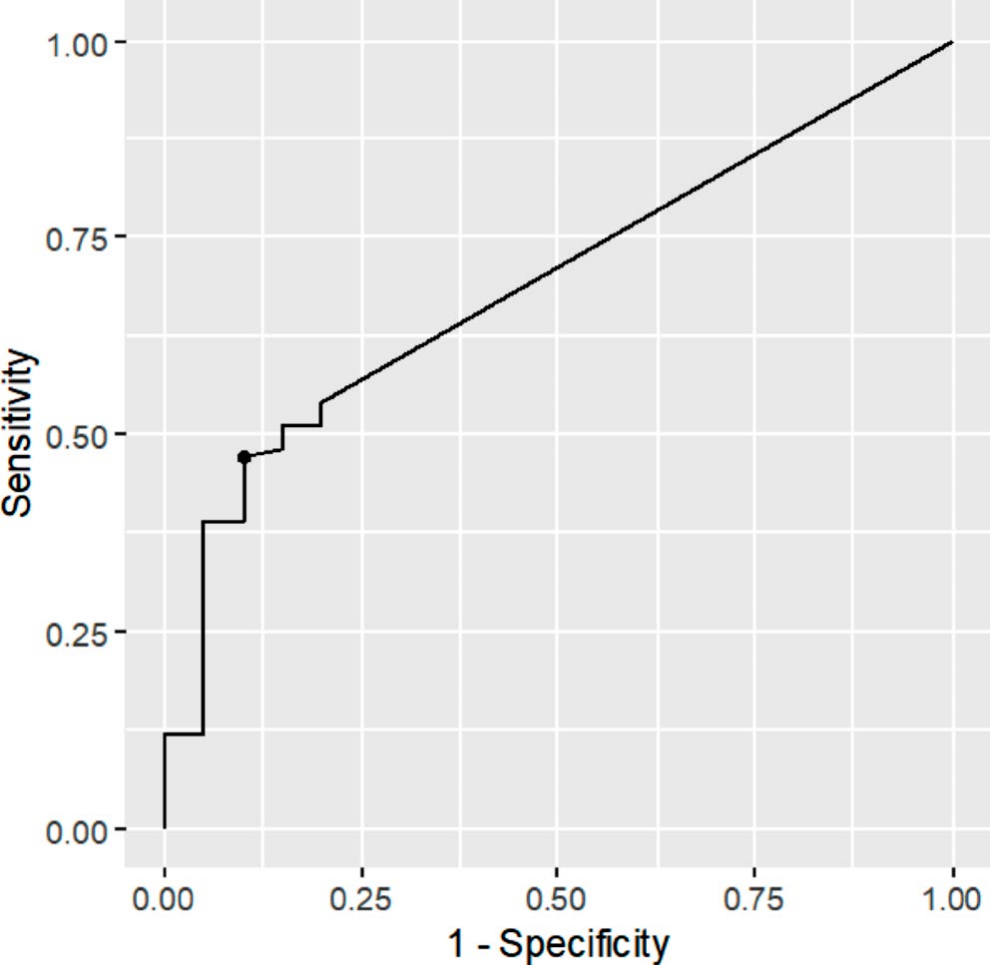
Receiver operating characteristic (ROC) analysis to predict a *Staphylococcus aureus* bacteremia (SAB) based on *S. aureus* NAAT detection in urine (Xpert^®^ SA Nasal Complete) using an optimal cut off Ct-value of 37.4. The area under the curve (AUC) of the corresponding receiver operating characteristic (ROC) analysis was 0.69

We calculated an optimal cut-off Ct-value of 37.4 (Youden-index = 0.37) for our sample set when maximizing the sum of sensitivity and specificity (Fig. S1). The area under the curve (AUC) of the respective ROC curve was 0.69 (Fig. 1).

In 25% (25/100) of SAB patients, we detected *S. aureus* by urine culture and in 47% (47/100) of patients (*p* = 0.002) by NAAT applying a Ct value of ≤ 37.4 (Table 1).

**Table 1:** Results of *Staphylococcus aureus* detection in urine culture and NAAT from urine in patients with SAB and non-SAB

*S. aureus* detection in urine		Bacteremia		
		SAB	Non-SAB	Total
Culture	Positive	25 (25%)	1 (5%)	26
	Negative	75 (75%)	19 (95%)	94
	Total	100	20	120
NAAT^a^	Positive	47 (47%)	2 (10%)	49
	Negative	53 (53%)	18 (90%)	71
	Total	100	20	120

^a^Ct values ≤ 37.4 were interpreted as positive

The detection of SAB by urine culture had a sensitivity of 25% (95%CI: 18–34%) and a specificity of 95% (95% CI: 76–99%) (Table 1). The detection of SAB by NAAT had a sensitivity of 47% (95%CI: 38–57%) and a specificity of 90% (95%CI: 70–97%, Table 1). The sensitivity for the molecular detection of *S. aureus* in urine was 53% (95%CI: 42–64%) if only patients were considered that had urine cultures collected between three days prior and up to one day after the collection of the positive blood culture. The specificity did not change if this stringent definition was applied (Table S1).

When excluding patients with indwelling catheters, the sensitivity for the molecular detection of *S. aureus* in urine was 49% (95%CI: 39–59%) and the specificity was 86% (95%CI: 60–96%, Table S2). Molecular and culture methods for the detection of *S. aureus* in urine showed a moderate agreement (80%, Cohens kappa: 0.55, Table 2).

**Table 2:** Concordance between NAAT and culture for the detection of *Staphylococcus aureus* in urine

		NAAT^a^		
		Positive	Negative	Total
Urine culture	Positive	25	1	26
	Negative	23	71	94
	Total	48	72	120

^a^Ct values ≤ 37.4 were interpreted as positive

Of 32 samples that were tested culture negative, but NAAT-positive (Ct value ≤ 37.4), 19 (59%) had a positive urine inhibitory test.

**Fig. 2:**
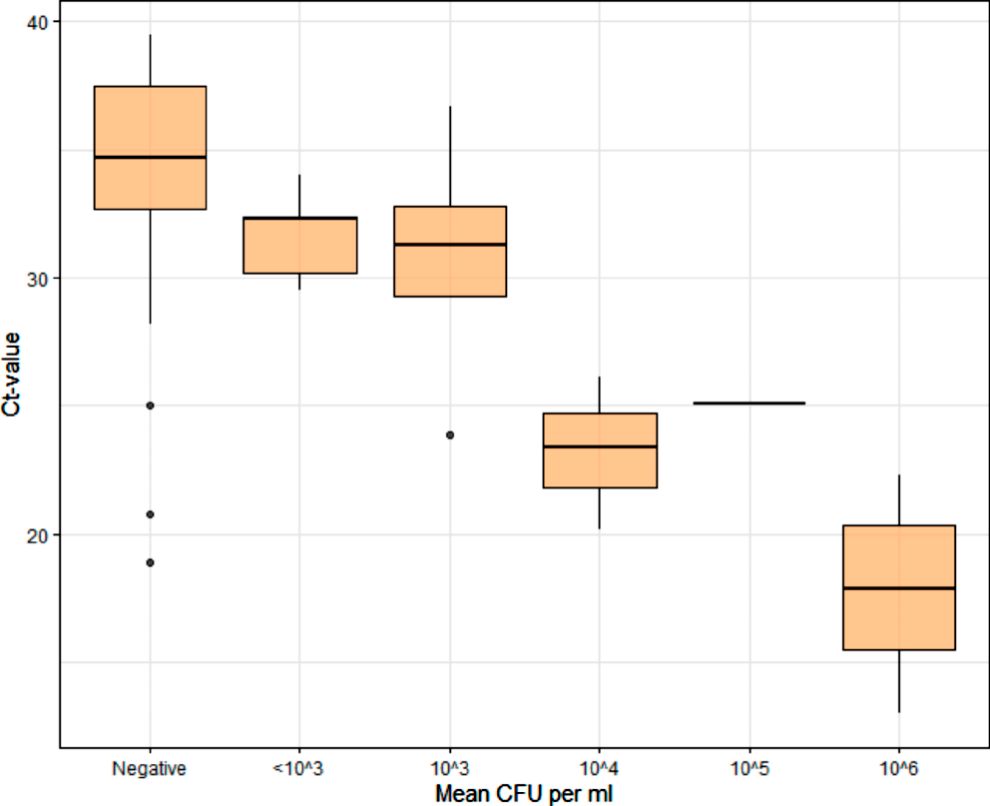
Correlation between Ct value of the NAAT detection of *Staphylococcus aureus* (Xpert^®^ SA Nasal Complete) and mean colony forming units [CFU] per ml of *S. aureus* on Columbia Blood Agar in urine. A total of 25 samples were included for which both the culture and a positive NAAT was available. Only one specimen had a *S. aureus* concentration of 10^5^ CFU/ml

Median Ct values for the detection of *S. aureus* in urine were higher in culture negative than in culture positive urine samples (34.6 vs. 23.9, Fig. 2). One patient with MRSA-bacteremia and MRSA-positive screening of the nose, throat and axilla had no detection of *S. aureus* in urine by NAAT. One patient with the detection of penicillin-susceptible *S. aureus* in blood and urine had a positive NAAT result for MRSA.

In the non-SAB group, 11 different species were detected. Bacteremia in non-SAB cases was mostly caused by *Escherichia coli* (*n* = 9) followed by *Staphylococcus epidermidis* (*n* = 2) and others (*n* = 9). Two non-SAB patients had a *S. aureus* detected in urine by NAAT and one of them was also culture positive for *S. aureus* (10^6^ CFU/ml). Both had nasal colonisation with *S. aureus*. The patient, who was also culture-positive, had primary skin disease and a superficial SSTI in the genital area.

The rate of NAAT detection of *S. aureus* in urine was lower in the non-SAB compared to the SAB group (5% [2/20] vs. 47% [47/100], *p* = 0.002).

Under the conditions of the study, the robustness/reproducibility of the test was 100% for positive and negative samples (Table S3). The LoD of the NAAT to detect SA in urine was 23 CFU/sample (Table S4). This corresponds to 354 CFU/ml of urine.

## Discussion

We compared the test performance of culture and a NAAT for the detection of *S. aureus* in urine from patients with bacteremia (SAB and Non-SAB). Main findings were a low-moderate sensitivity (47%) and high specificity (90%) of the urine NAAT to identify patients with SAB. The sensitivity increased (53%) if only patients were considered that have a urine culture collected three days prior and up to one day after the collection of the positive blood culture. Later collections reduce sensitivity e.g. if the patient is already on empiric antibiotics. We saw that culture negative, but NAAT-positive urine samples had a positive inhibitory test in more than one-half of the cases. We conclude that antibacterial substances in urine samples (i.e. antibiotics) are frequent and might inhibit culture growth of *S. aureus.*

These findings support the value of detecting *S. aureus* in urine as early as possible after presentation/admission in the emergency department.

The relevance of bacterial detection in urine up to three days prior to detection of bacteremia remains unknown as the number of patients with a urine culture sampled on the days before the blood culture was small in our cohort (*n* = 6, 67% NAAT positive).

The test cartridges were used to analyse the urine samples with a rapid and easy-to-handle method. For the rapid diagnosis of invasive *Legionella pneumophila* and pneumococcal infections, antigen-based immunochromatographic tests from urine are integrated into routine diagnostics since many years. These tests have a good sensitivity (95–97%) and specificity (95–99%) according to the manufacturer to diagnose invasive infections (e.g. pneumonia). In contrast, the test performance of NAAT from urine to detect SAB has a much lower sensitivity in our study. When performing the NAAT, we followed the proposed method for MRSA screening from nasal swabs. We calculated a mean volume of 65 µl [49.7–83.4 µl] that can be squeezed out of the swabs and was therefore transferred into the elution buffer of the cartridge test. We suggest for further studies a higher volume of urine (e.g. 100 µl) directly transferred to the test cartridge possibly achieving a higher sensitivity. The target gene in Xpert^®^ SA Nasal Complete Assay for the detection of *S. aureus* is *spa*, which can vary in size between different *S. aureus spa* types and is located as a single copy in the genome. Other (multi-copy) genes or antigens with high expression levels might therefore be alternative targets to detect *S. aureus* in urine of patients with SAB. Such a urine antigen test could be used as a Point-of-Care-Testing (POCT) that would enable the use of narrow spectrum antimicrobial agents in septic patients.

Taking into account the low-moderate sensitivity, further research would be needed to assess whether detection of *S. aureus* in urine has a clinical benefit. For that purpose, time to optimal therapy and treatment outcomes could be compared between patients (e.g. matched for age, sex, comorbidities), whose antimicrobial therapy was guided by NAAT from urine vs. blood culture, multiplex-PCR or MALDI-TOF MS from short sub-cultures on solid medium.

We had one false-positive MRSA detection in NAAT, which was not confirmed by culture of blood and/or urine. The manufacturer of the NAAT reports a specificity of 97.9% for the detection of MRSA. This minor error would lead to a less effective initial therapy (e.g. vancomycin) for susceptible isolates.

According to the manufacturer, the LoD for *S. aureus* in nasal swabs is 93.7 (95% CI 75.5 -137.8) CFU/sample. Compared to nasal swabs, we estimated a lower LoD (23 CFU/sample) for the detection of *S. aureus* from urine. One reason for this difference is most likely the difference in calculating the LoD. Since the calculated CFU/sample of a dilution series only gives an inaccurate estimate of the real CFU/sample, we decided to plate the bacterial suspension of the dilution series and use the actual bacterial counts to calculate the LoD.

Our study has limitations. First, urine samples positive for *S. aureus* by NAAT in patients with and without SAB might be due to perineal colonisation or contamination of the urine vial during the collection of the specimen. Second, since the study design is cross sectional, we cannot state if SAB was secondary to a urinary tract infection or vice versa. We rate the risk as low, as true *S. aureus* urinary tract infections in our setting are uncommon because we detect *S. aureus* in urine in only 1.4–1.9% of all positive cultures from clinical samples [14]. In addition, the urinary tract as the primary source of SAB is infrequent (3.2%, *n* = 132/4181 SAB patients) [26]. Third, the Xpert^®^ SA Nasal Complete detects *S. aureus* by the amplification of *spa*. False negative results are possible due to deletions in *spa* [33]. Fourth, due to high costs of the test cartridges, the approach of this study might not be suitable for all cases in routine diagnostics. Fifth, due to the small number of non-SAB cases, the specificity could only be calculated with a low degree of accuracy.

## Conclusions

Using NAAT detection of *S. aureus* in urine we were able to identify patients with SAB with a low-moderate sensitivity (47%) and a high specificity (90%) in our population. This warrants further evaluation of urine rapid tests for the detection of SAB.

## Electronic supplementary material

Below is the link to the electronic supplementary material.

Supplementary Material 1

## Data Availability

No datasets were generated or analysed during the current study.

## Abbreviations

AMR: Antimicrobial resistance
AUC: Area under the curve
CFU/ml: Colony forming units per ml
LoD: Limit of detection
MALDI-TOF MS: Matrix-assisted laser desorption/ionization time-of-flight mass spectrometry
MRSA: Methicillin-resistant *Staphylococcus aureus*
NAAT: Nucleic acid amplification testing
PCR: Polymerase chain reaction
POCT: Point-of-Care-Testing
ROC: Receiver operating characteristic
SAB: *Staphylococcus aureus* bacteremia
SABU: *Staphylococcus aureus* bacteriuria
