# Supplementary material for: Molecular detection of Staphylococcus aureus in urine in patients with S. aureus bacteremia: an exploratory study
Source: Eur J Clin Microbiol Infect Dis. 2024 Oct 31;44(1):37–43. doi: 10.1007/s10096-024-04969-7 (PMC11739235; doi:10.1007/s10096-024-04969-7)
Supplement: Supplementary file 1 — Supplementary Material 1 [file 10096_2024_4969_MOESM1_ESM.docx]

**Tables**

Table S1: Molecular detection of *S. aureus* in urine depending on the time interval between the collection of the urine and blood culture

|  | **time difference of the collection of the urine compared to the collection of the blood culture** | | | | | | |
| --- | --- | --- | --- | --- | --- | --- | --- |
|  | **-3d** | **-2d** | **-1d** | **0d** | **+1d** | **+2d** | **+3d** |
| **patients with SAB** | 1 | 2 | 3 | 54 | 19 | 10 | 11 |
| **patients with SAB and a positive NAAT^a^** | 0 | 2 | 2 | 28 | 10 | 3 | 2 |

^a^Ct values ≤37.4 were interpreted as positive.

Table S2: Subgroup sensitivity analysis in patients with and without indwelling catheters

|  | **Number of results test/blood culture** | | | | **Sensitivity TP/(TP+FN)**  **(95% CI)** | **Specificity TN/(TN+FP)**  **(95% CI)** |
| --- | --- | --- | --- | --- | --- | --- |
|  | **TP** | **FP** | **FN** | **TN** |  |  |
| **Patients including those with indwelling catheters (n=120)** | | | | | | |
| Urine culture | 25 | 1 | 75 | 19 | 25% (18-34) | 95% (76-99) |
| NAAT^a^ | 47 | 2 | 53 | 18 | 47% (38-57) | 90% (70-97) |
| **Patients excluding those with indwelling catheters (n=102)** | | | | | | |
| Urine culture | 23 | 1 | 65 | 13 | 26% (18-36) | 93% (69-99) |
| NAAT^a^ | 43 | 2 | 45 | 12 | 49% (39-59) | 86% (60-96) |

^a^Ct values ≤37.4 were interpreted as positive.TP=True positive, FP=False positive; FN= False negative; TN=True negative.

Table S3: Validation of the Xpert® SA Nasal Complete for the use of urine as a matrix. Target gene: spa.

| **Sample** | **Day 1**  **Ct value** | **Day 2**  **Ct value** | **Day 3**  **Ct value** | **Robustness (Inter-Assay precision)** | **Target value** |
| --- | --- | --- | --- | --- | --- |
| No_76 | 18.7 | 20.4 | 19.6 | 100% | 100% |
| No_99 | 0.0 | 0.0 | 0.0 | 100% | 100% |

Table S4: Serial dilution (*Staphylococcus aureus* ATCC 29213) to calculate the limit of detection of the Xpert® SA Nasal Complete for the use of urine as a matrix. Target gene: *spa.*

| **Day 1** | | | **Day 2** | | | **Day 3** | | |
| --- | --- | --- | --- | --- | --- | --- | --- | --- |
| CFU  /sample | PCR result | Ct value^a^ | CFU  /sample | PCR result | Ct value^a^ | CFU  /sample | PCR result | Ct value^a^ |
| 0 | neg | 0.0 | - | - | - | - | - | - |
| 9 | neg | 0.0 | - | - | - | - | - | - |
| 23 | pos | 35.1 | 23 | pos | 33.7 | 23 | pos | 35.3 |
| 59 | pos | 35.0 | - | - | - | - | - | - |
| 117 | pos | 33.2 | - | - | - | - | - | - |

^a^Ct values ≤37.4 were interpreted as positive. neg = negative; pos=positive

**Figures**


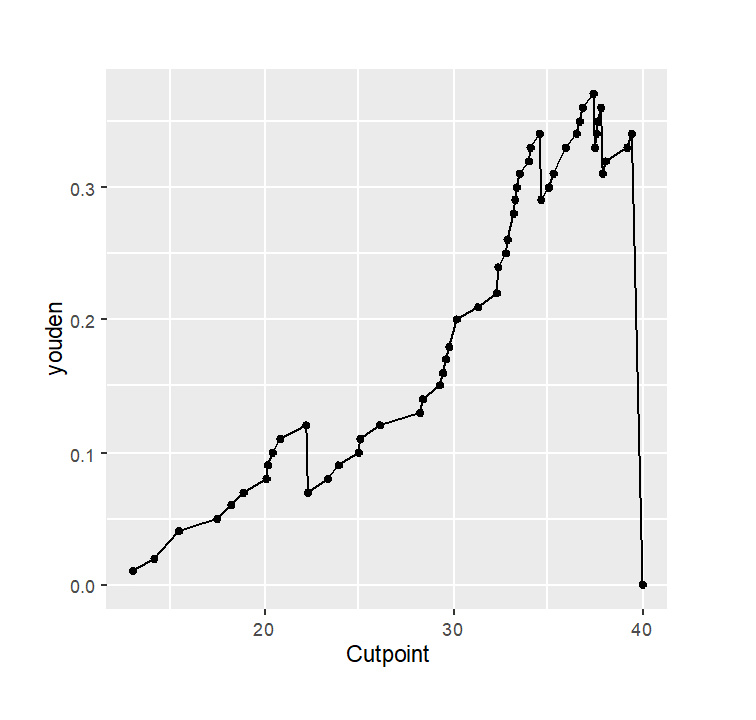


Figure S1: Calculation of the optimal cut-off Ct value to predict a *Staphylococcus aureus* bacteremia (SAB) based on *S. aureus* NAAT detection in urine (Xpert^®^ SA Nasal Complete). When maximizing the sum of sensitivity and specificity for the prediction of SAB, a cut-off Ct value of 37.4 (vertical line) had the highest Youden-Index (0.37).
